# Global Research on Cognitive Frailty: A Bibliometric and Visual Analysis of Papers Published during 2013–2021

**DOI:** 10.3390/ijerph19138170

**Published:** 2022-07-04

**Authors:** Zhaozhao Hui, Xiaoqin Wang, Ying Zhou, Yajing Li, Xiaohan Ren, Mingxu Wang

**Affiliations:** 1 School of Public Health, Health Science Center, Xi’an Jiaotong University, 76 Yanta West Road, Xi’an 710061, China; huizzjoy@mail.xjtu.edu.cn (Z.H.); liyajing1119@stu.xjtu.edu.cn (Y.L.); 2 School of Nursing, Health Science Center, Xi’an Jiaotong University, 76 Yanta West Road, Xi’an 710061, China; wangxiaoqin@mail.xjtu.edu.cn (X.W.); rxh3119315241@stu.xjtu.edu.cn (X.R.); 3 Office of Cadre Health Care, The First Affiliated Hospital of Xi’an Jiaotong University, 277 Yanta West Road, Xi’an 710061, China; 7204@xjtufh.edu.cn

**Keywords:** cognitive frailty, older adult, bibliometric analysis, CiteSpace

## Abstract

This study analyzed the current status, hotspots, and emerging trends of global research on cognitive frailty, in order to provide new research ideas for researchers. Articles and reviews related to cognitive frailty, published from 2013 to 2021, were retrieved from the Web of Science Core Collection (WoSCC) database on 26 November 2021. CiteSpace 5.8.R3 was employed for data analyses. A total of 2077 publications were included. There has been a rapid growth of publications on cognitive frailty research since 2016. The United States, Italy, England, and Australia have been the leading research centers of cognitive frailty; however, China has also recently focused on this topic. The National Center for Geriatrics and Gerontology, and Shimada H. were found to be the most prolific institution and author, respectively. Co-citation analysis identified 16 clusters, of which the largest was cognitive frailty. The keywords which occurred most frequently were “older adult”, followed by “cognitive impairment”, “frailty”, “risk”, “dementia”, “prevalence”, “mortality”, “health”, and “Alzheimer’s disease”. Burst keyword detection revealed a rising interest in cognitive frailty models. By analyzing these publications from recent years, this study provides a comprehensive analysis of cognitive frailty research.

## 1. Introduction

Frailty is a common geriatric syndrome, in which there is an increase in an individual’s vulnerability to stressors, as a consequence of the reduced capacity of different physiological systems, characterized by diminished strength, endurance, and reduced physiologic function [1]. Among older people, physical frailty and cognitive impairment often co-occur and predict the onset of each other [2,3,4], as they can share the same mechanisms, such as chronic inflammation, impaired hypothalamic–pituitary axis stress response, imbalanced energy metabolism, mitochondrial dysfunction, oxidative stress, and neuroendocrine dysfunction [5]. In 2013, the consensus group of the International Academy on Nutrition and Aging and the International Association of Gerontology and Geriatrics (IANA/IAGG) officially defined cognitive frailty as a syndrome in older adults with evidence of both physical frailty and cognitive impairment with the absence of Alzheimer’s disease or any other dementias [6].

As the interest in healthy aging increases, a better understanding of cognitive frailty may help maintain the functional ability of older adults [7]. Since 2013, cognitive frailty has attracted much attention from health professionals and researchers. While the psychometrically appropriate measures for this novel construct need further investigation, cognitive frailty is mainly diagnosed with clinical evaluations including MMSE, executive tests, gait speed, grip strength, weight loss, and psychological markers [8]. A recent systematic review found that the prevalence of cognitive frailty reaches 9% in community-dwelling older adults, and has increased in recent years [9]. Evidence showed that a variety of socio-demographic factors and health conditions can increase the risk of cognitive frailty, such as advanced age, low schooling, comorbidity, malnutrition, low social participation, sedentary lifestyle, and insomnia [10,11]. Moreover, many studies have revealed that cognitive frailty can significantly increase the risk of dementia, mortality [10,12,13], falls [14,15], and disability [16] in older adults.

Given the increasing prevalence and adverse health outcomes, it is necessary to pour considerable efforts into this research field. Understanding the dynamics of a research front is essential for researchers to be able to identify hotspots and emerging trends in the body of scientific knowledge [17]. Although several reviews have been conducted for cognitive frailty [9,10,12,13,18,19], no studies have previously attempted to analyze the development of cognitive frailty research since its definition was officially proposed. Bibliometric analysis enables researchers to unpack the evolutionary nuances of a specific field while shedding light on the emerging areas in that field by making sense of large volumes of unstructured data in rigorous ways [20]. Based on bibliometric analysis, this study aimed to systematically investigate the status, hotspots, and emerging trends/frontiers of global research on cognitive frailty.

## 2. Methods

### 2.1. Data Source and Search Strategy

The Web of Science Core Collection (WoSCC) database, which contains the world’s leading scholarly journals, was selected as the data source for this study. The literature related to cognitive frailty was searched from 2013 to 26 November 2021. The search terms and strategy were: (“cognitive frailty”) OR ((“cognitive decline” OR “cognitive impairment”) AND (frail*)). To examine the effectiveness of the search results, one researcher assessed the relevance, to this study, of the top 100 most recently published articles. The results showed that 86% were related to cognitive frailty, indicating that the search terms and strategies were appropriate.

### 2.2. Inclusion and Exclusion Criteria

Inclusion criteria were: (a) peer-reviewed original articles on cognitive frailty; (b) reviews related to cognitive frailty; (c) articles retrieved from the WoSCC database; (d) articles published from 2013 to 2021; and (e) articles published in English. Articles collected by hand, repeated publications, conference abstracts, and book chapters were excluded from the bibliometric analysis. Two researchers independently screened the literature according to the inclusion and exclusion criteria. Disagreement was resolved by consulting a third researcher.

### 2.3. Data Analysis and Visualization

GraphPad Prism 9 and CiteSpace 5.8.R3 were used to analyze the included articles. The GraphPad Prism software (GraphPad Software, San Diego, CA, USA) was adopted for making bar and line charts. CiteSpace was used to conduct the bibliometric analysis, including collaboration analysis, document co-citation analysis, and keyword co-occurrence analysis. Different nodes in visualization knowledge maps represented elements such as country, institution, author, or a cited reference; links between the nodes represented relationships of collaboration, co-occurrence, or co-citations; the color of the nodes and lines represented different years. Centrality measured the extent to which a node was part of a path that connected an arbitrary pair of nodes in the network. CiteSpace highlighted, with purple rings, nodes with high centrality, of which the thickness indicated how strong their centrality was. The parameters of CiteSpace were set as follows: (1) time-slicing from January 2013 to December 2021, years per slice = 1; (2) term source = title/abstract/author keywords/keywords plus; (3) node types = country/author/institution/reference/keyword; (4) select top 10% of most cited or occurred items from each slice (except country collaboration analysis); (5) pruning = none; (6) visualization: cluster static, show merged network. Modularity Q > 0.3 meant that the network was reasonably divided into loosely coupled clusters, and the mean Silhouette score > 0.7 indicated that the homogeneity of the clusters on average was significant [21].

## 3. Results

### 3.1. Publication Years and Journals

**Figure 1:**
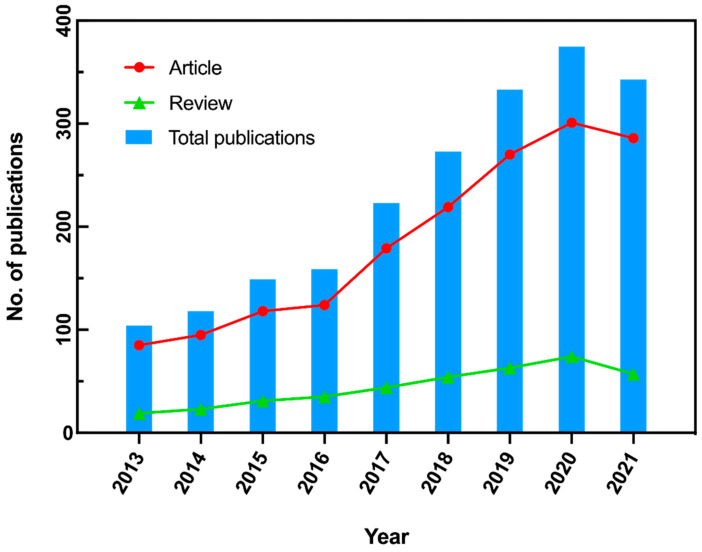
Number of publications on cognitive frailty in the Web of Science Core Collection database from 2013 to 2021. The number of original articles published per year shows a slow growth and then a noticeable rise, while the review exhibits a steady upward trend.

A total of 2077 publications were retrieved from the WoSCC database. Among these publications, 1677 were original articles (80.7%) and 400 were reviews (19.3%). The year-wise distribution of publications on cognitive frailty from 2013 to 2021 is shown in Figure 1. The red points represent the number of original articles published per year, and the blue bar graphs demonstrate the number of total publications, both indicating a slow growth and then a noticeable rise, except for the final year. Of note, more than 200 papers were annually published since 2017. The green triangles illustrate the annually published reviews, exhibiting a steady upward trend during the period of 2013–2020. These results indicate that cognitive frailty is receiving increased attention in the research field, and that more relevant studies are being performed.

Elsevier published the largest number of papers on cognitive frailty from 2013 to 2021 (*n* = 510). Regarding journals, four published at least 50 publications on cognitive frailty, of which BMC Geriatrics was the most prolific (*n* = 75). The top 10 journals that published the largest number of papers on cognitive frailty contributed to 23.11% of the total publications, and their impact factors ranged from 2.730 to 6.053 (Table 1). Among them, Journals of Gerontology Series A: Biological Sciences and Medical Sciences, ranking No. 7, had the highest impact factor (6.053).

### 3.2. Collaboration Analysis

#### 3.2.1. Country Collaboration Analysis

**Figure 2:**
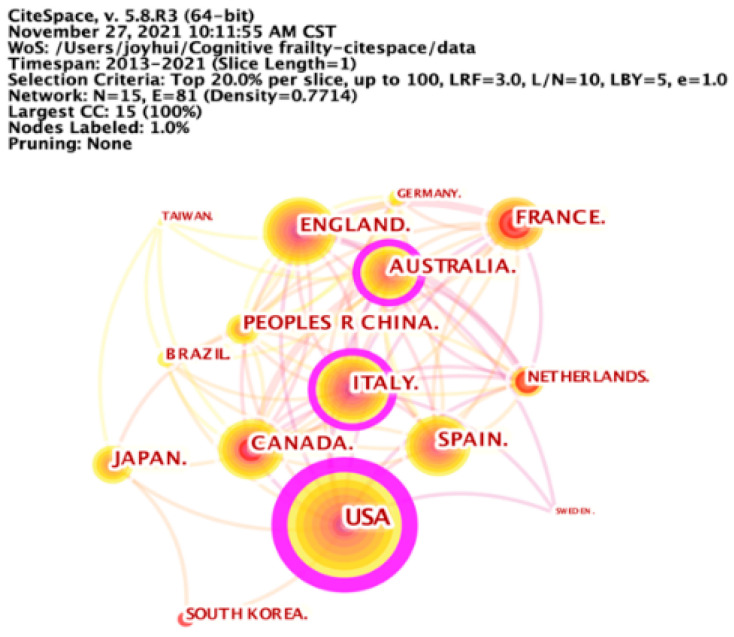
Collaboration network of countries/regions. The United States, Italy, and Australia have made great contributions to the research field of cognitive frailty, and have closely cooperated with other countries/regions.

We selected the “Country” node type to analyze the degree of collaboration among countries/regions in cognitive frailty research, in which the top 20% of most-occurred items were selected from each slice (Figure 2). A total of 87 countries/regions contributed to research on cognitive frailty from 2013 to 2021. The United States published the largest number of papers in this research area (*n* = 505), accounting for 24.31% of the publications, followed by Italy (*n* = 240) and England (*n* = 192). The top three countries published 937 papers on cognitive frailty, accounting for 45.11% of the publications. With high centrality, the United States (0.35), Italy (0.13), and Australia (0.13) made great contributions to this research field, and closely cooperated with other countries/regions. According to the burst detection, China was found to be the most recently emerging country to have focused on cognitive frailty research.

#### 3.2.2. Institution Collaboration Analysis

**Figure 3:**
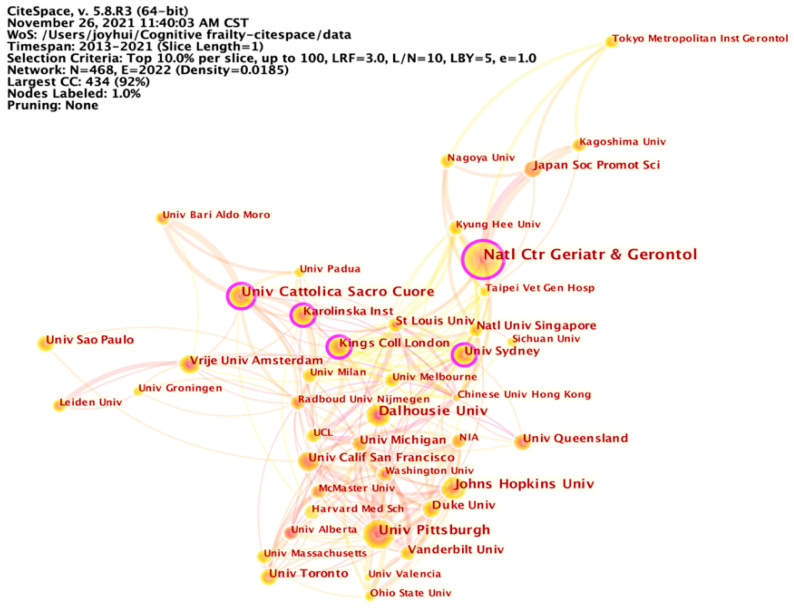
Collaboration network of institutions. Università Cattolica del Sacro Cuore, King’s College London, the University of Sydney, the Karolinska Institute, and the National Center for Geriatrics and Gerontology closely cooperated with other institutions in cognitive frailty research.

By selecting the “Institution” node type, we analyzed the degree of collaboration among institutions in this research field. Each node represented an institution that had published more than 15 papers related to cognitive frailty. The National Center for Geriatrics and Gerontology made the greatest contribution to this research topic, having published 69 articles or reviews (3.13%), followed by Johns Hopkins University (*n* = 42, 1.90%) and Dalhousie University (*n* = 40, 1.81%). As for the centrality, Università Cattolica del Sacro Cuore (0.19), King’s College London (0.13), the University of Sydney (0.12), the Karolinska Institute (0.12), and the National Center for Geriatrics and Gerontology (0.11) represented the major turning points, acting as bridges linking other institutions. Furthermore, the greatest number of bursts in cognitive frailty research was attributed to the University of Alberta (4.56), which is a public research university located in Canada. Figure 3 presents the collaboration network of institutions publishing papers on cognitive frailty during 2013 to 2021.

#### 3.2.3. Author Collaboration Analysis

**Figure 4:**
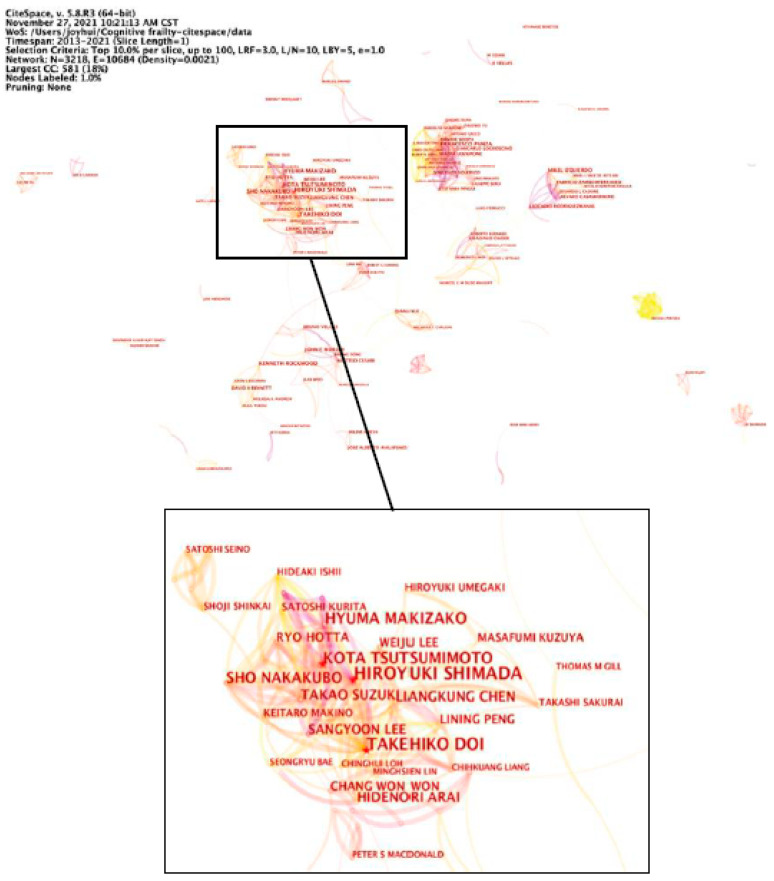
Collaboration network of authors. Shimada H., Doi T., and Tsutsumimoto K. were the three most prolific authors of cognitive frailty research.

A scientific co-authorship network can provide information on influential authors and potential collaborators, and can help researchers to establish collaborative relationships. The node type of “Author” was selected for conducting the author collaboration analysis. Figure 4 illustrates the collaboration network of authors who published at least three papers related to cognitive frailty from 2013 to 2021; the centrality of all authors was less than 0.1.

Regarding productivity, Shimada H. was identified as the most prolific author with 37 articles, followed by Doi T. (*n* = 28) and Tsutsumimoto K. (*n* = 27). These three authors all come from Japan, and collaborated with each other. Of them, Shimada H. had the highest citation impact of publications (H index = 41).

#### 3.2.4. Document Co-Citation Analysis

**Figure 5:**
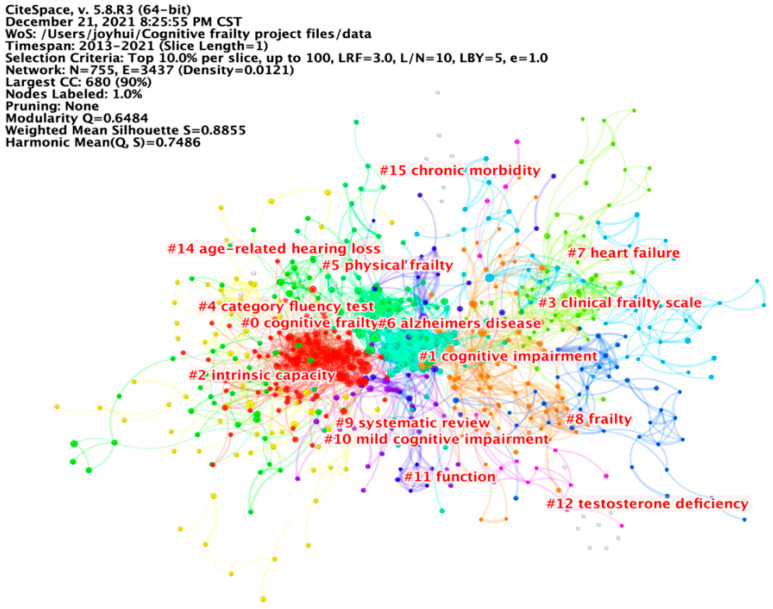
Document co-citation network in cognitive frailty research. A total of 69,148 valid references were extracted, and a network consisting of 755 nodes and 3437 links was visualized. The largest three clusters were cognitive frailty, cognitive impairment, and intrinsic capacity.

By selecting “Reference” as the node type, a document co-citation analysis was conducted. A total of 69,148 valid references were extracted, and a network consisting of 755 nodes and 3437 links was visualized (Figure 5). The network was divided into 16 clusters, which were automatically labeled by choosing keyword terms as the labeling source, and log-likelihood ratio (LLR) as the standard algorithm. The largest cluster (#0) was cognitive frailty, followed by cognitive impairment (#1), intrinsic capacity (#2), clinical frailty scale (#3), category fluency test (#4), and physical frailty (#5). The modularity Q was 0.648, and the mean Silhouette score was 0.886, suggesting that the cluster results were reasonable.

Table 2 presents 15 representative references in the field of cognitive frailty. Nos. 1–10 were cited most. Nos.1 & 11 had the highest centrality, indicating close interrelationships with other references. Nos. 1–4, 6, and 12–15 had the strongest burst strength, and could be regarded as the research frontiers and trends on cognitive frailty. Of the three most cited works, ‘Frailty in elderly people’ [22] was a review focused on frailty, including its definition and presentations, pathophysiology, models, instrumentation, and interventions; ‘Cognitive frailty: rational and definition from an (I.A.N.A./I.A.G.G.) International Consensus Group’ provided the first definition of a “Cognitive Frailty” condition in older adults [6]; and ‘Frailty and cognitive impairment—a review of the evidence and causal mechanisms’ reviewed the evidence for an association between physical frailty and cognitive impairment, and outlined some of the mechanisms that potentially underpin this relationship, from brain neuropathology and hormonal dysregulation to cardiovascular risk and psychological factors [2].

#### 3.2.5. Keyword Co-Occurrence Analysis

**Figure 6:**
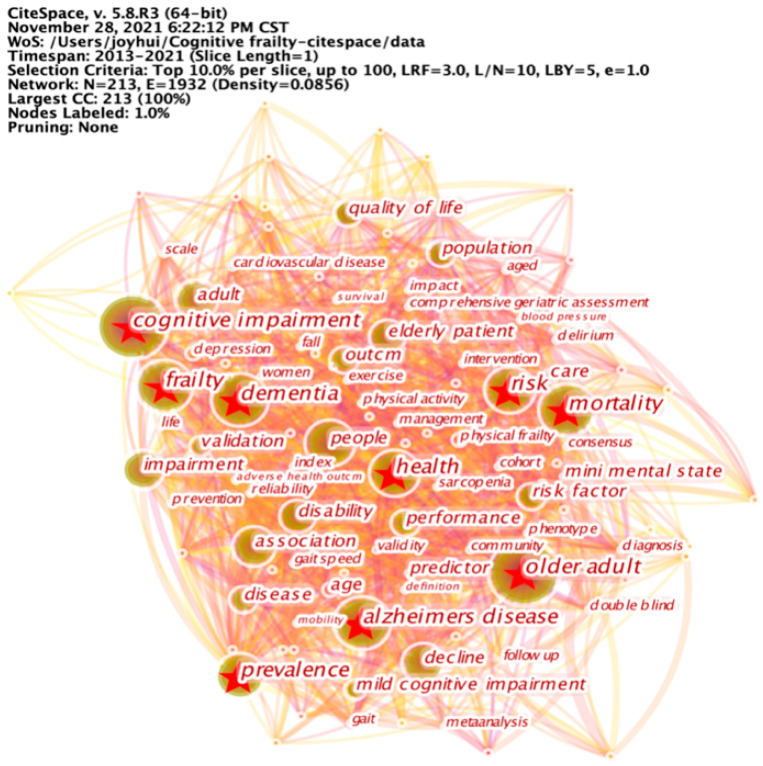
Network of co-occurring keywords. The most frequently occurring keyword in cognitive frailty research was “older adult”, followed by “cognitive impairment”, “frailty”, “risk”, “dementia”, “prevalence”, “mortality”, “health”, and “Alzheimer’s disease”.

To illustrate the hotspots of cognitive frailty research, we conducted a keywords co-occurrence analysis by changing the node type to “Keyword”. To avoid potential misunderstanding, some similar keywords were combined. For example, “older people”, “elderly people”, “older adult”, and “older person” were merged into “older adult”; “cognitive decline” and “cognitive impairment” were merged into “cognitive impairment”. The network of co-occurring keywords, with 213 nodes and 1932 links, is shown in Figure 6. Bigger nodes indicate higher co-occurrence frequency, and the links reflect the co-occurrence relationship. The most frequently occurring keyword was “older adult” (count = 591), followed by “cognitive impairment”, “frailty”, “risk”, “dementia”, “prevalence”, “mortality”, “health”, and “Alzheimer’s disease”.

**Figure 7:**
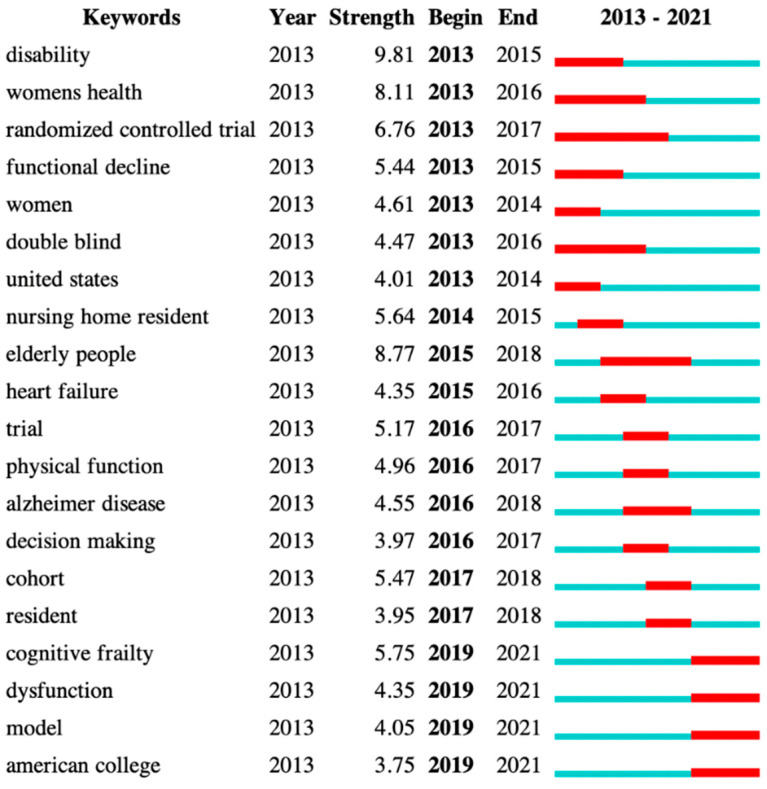
20 keywords with the strongest citation burst. “Disability” shows the highest burst strength, while “cognitive frailty”, “dysfunction”, “model”, and “American college” were the most recent citation bursts.

Burst keywords were detected, to analyze the hotspots and frontier of cognitive frailty research. We identified the top 20 keywords with the strongest citation burst (Figure 7). Of them, “disability” showed the highest burst strength, reaching 9.61. “Cognitive frailty”, “dysfunction”, “model”, and “American college” were keywords with recent citation bursts, and “randomized controlled trial” achieved the longest burst duration from 2013 to 2017.

## 4. Discussion

To the best of our knowledge, this is the first study to systematically analyze the status, hotspots, and emerging trends/frontiers of global research on cognitive frailty through bibliometric and visual analysis. Our results show that cognitive frailty has attracted increasing research interest since 2013, and that this trend is projected to continue, as indicated by the increase in the number of annual publications on this research topic. By analyzing the sources of the publications, it can be inferred that the global research on cognitive frailty has formed a core group of journals, as nearly a quarter of the papers were published in the top 10 journals.

The United States, Italy, England, and Australia are the leading countries in cognitive frailty research, as evidenced by the large number of publications and the high centrality in the country collaboration network. Moreover, more than half of the top 10 prolific institutions were from these countries. These results are roughly consistent with previous bibliometric studies among older people [33,34]. When conducting burst detection, China was found to be the most recently emerging country to focus on cognitive frailty research. The results of this study indicate, to a certain extent, that health care practitioners and researchers from China have begun to focus on cognitive frailty research. Cognitive frailty could increase the risk of adverse health outcomes among older people, such as dementia, falls, disability, and mortality [10,12,13,14,15,16], which may further increase the health care expenditure of the sufferers and threaten their quality of life. As China has the world’s largest population of older people, Chinese scholars have suggested continuing to focus on cognitive frailty research for the next few years, especially in collaboration with productive institutions from the aforementioned countries. In addition, this study found that the most three prolific authors in cognitive frailty research were Shimada H., Doi T., and Tsutsumimoto K., who can be considered potential collaborative authors.

In terms of structure and hotspots within the knowledge domain of cognitive frailty, the predictive value of cognitive frailty on health-related outcomes is a popular topic [13,16,27,35,36,37,38,39]. Apart from the risk of dementia, mortality, and disability, some studies focused on surgical patients, and examined the predictive effect of combined preoperative assessment of frailty and cognitive function for postoperative complications (e.g., delirium, long hospital stay) [36,37]. Another popular research topic is an investigation of the prevalence of cognitive frailty in people with different characteristics, including nursing home residents [40]. The third popular topic is the biomarkers of physical and cognitive function among older people, such as testosterone deficiency [41]. Moreover, researchers have paid much attention to preventive interventions/programs for cognitive frailty, including resistance exercise training [42], mHealth brisk walking intervention [43], Baduanjin [44], and multi-domain interventions [45,46,47]. 

Regarding representative references for cognitive frailty research, the paper ‘Frailty in elderly people’, published by Clegg, et al., seems to have laid the foundation for this research area [22], as evidenced by the highest frequency of citation. Another two landmark publications entitled ‘Cognitive frailty: Rational and definition from an (I.A.N.A./I.A.G.G.) International Consensus Group’ [6], and ‘Frailty and cognitive impairment—A review of the evidence and causal mechanisms’ [2] served as pivotal points of cognitive frailty research, as they officially defined cognitive frailty and outlined some mechanisms that underpin the interaction between physical frailty and cognitive decline, respectively. Additionally, an Italian longitudinal study published in 2017, which estimated the predictive role of reversible cognitive frailty on dementia and all-cause mortality [27], and a comprehensive review of different cognitive frailty models and health- and cognitive-related outcomes, published in 2018 [31], were identified as two references of the most recent bursts. These landmark references have laid a solid theoretical foundation for future research in this research field.

For burst keyword detection, those with recent citation bursts suggest the research frontier of cognitive frailty, which needs more attention from elderly practitioners and researchers in the coming years. For example, although different cognitive frailty models (e.g., the potentially reversible cognitive frailty model) have been proposed [42,48], a new model from a psychosocial and behavioral perspective could be examined in future studies, as evidence that cognitive frailty is associated with various psychosocial and behavioral factors [48]. In addition, the mechanisms that potentially underpin the relationship between physical frailty and cognitive impairment and cost-effective interventions should be investigated in future studies. Another important burst keyword is “disability”, which showed the highest strength, indicating that the association between cognitive frailty and disability may be extensively studied. It is worth noting that “intrinsic capacity”, which was proposed by the World Health Organization in 2015 [49], was identified as a large cluster for cognitive frailty research. Disability is traditionally measured by the ability to perform activities of daily living (ADL) and instrumental ADL (IADL); however, it has been demonstrated that intrinsic capacity could predict declining performance in both IADLs (β = −0.324) and ADLs (β = −0.227) [50]. Moreover, intrinsic capacity is a multidimensional indicator related to the individual’s functional status, whose follow-up over time may be useful for healthy aging [49,51]. Future studies are therefore suggested, to investigate the levels as well as the trajectories of intrinsic capacity in older people with cognitive frailty.

Although the results of this study have provided valuable information to future cognitive frailty researchers and practitioners, limitations should be considered when interpreting the findings. Given the rapid development of cognitive frailty research, this study does not include works published in recent months. Furthermore, this study searched the publications of the WoSCC database only. It was impossible to include all papers related to cognitive frailty. However, the WoSCC database is considered the most authoritative one, which contains the world’s leading scholarly journals. While this study is the first bibliometric analysis of cognitive frailty research, scholars who want to deeply delve into this field are advised to search other databases, to identify more relevant publications.

## 5. Conclusions

This study provides a comprehensive bibliometric analysis of cognitive frailty research. A variety of visualized networks offer an in-depth understanding of the countries/regions, institutions, authors, hotspots, and research frontiers. For cognitive frailty researchers and practitioners, this study provides accurate information regarding potential collaborative authors and institutions for reference. Moreover, this study also identifies the hotspots and frontiers in this research area, which can provide guidance for future studies.

## Figures and Tables

**Table 1:** Top 10 most prolific journals.

Journal	No. of Publications (%)	IF ^a^	JCR ^®^ Category
BMC Geriatrics	75 (3.61)	3.921	Geriatrics & Gerontology (Q2) Gerontology (Q1)
2. Journal of The American Medical Directors Association	62 (2.99)	4.669	Geriatrics & Gerontology (Q2)
3. Journal of Nutrition Health Aging	61 (2.94)	4.075	Geriatrics & Gerontology (Q2) Nutrition & Dietetics (Q2)
4. Geriatrics & Gerontology International	52 (2.50)	2.730	Geriatrics & Gerontology (Q3) Gerontology (Q2)
5. Archives of Gerontology and Geriatrics	46 (2.22)	3.250	Geriatrics & Gerontology (Q3)
6. Journal of The American Geriatrics Society	44 (2.12)	5.562	Geriatrics & Gerontology (Q1) Gerontology (Q1)
7. Journals of Gerontology Series A: Biological Sciences and Medical Sciences	41 (1.97)	6.053	Geriatrics & Gerontology (Q3) Gerontology (Q1)
8. Aging Clinical and Experimental Research	33 (1.59)	3.638	Geriatrics & Gerontology (Q3)
9. Clinical Interventions in Aging	33 (1.59)	4.458	Geriatrics & Gerontology (Q2)
10. Journal of Alzheimer’s Disease	33 (1.59)	4.472	Neurosciences (Q2)

Notes: IF = impact factor; JCR = Journal Citation Reports. ^a^ Data from the 2020 edition of Journal Citation Reports.

**Table 2:** Fifteen representative references in terms of citations, centrality, and bursts.

No.	Count	Centrality	Strength	Reference	Year	Begin	End
1	126	0.12	26.58	Clegg, et al. [22]	2013	2015	2018
2	109	0.01	27.16	Kelaiditi, et al. [6]	2013	2016	2018
3	92	0.02	18.96	Robertson, et al. [2]	2013	2015	2018
4	83	0.00	18.00	Morley, et al. [1]	2013	2015	2018
5	73	0.06	7.54	Feng, et al. [23]	2017	2018	2021
6	59	0.03	15.23	Shimada, et al. [24]	2013	2015	2018
7	58	0.01	10.26	Ruan, et al. [25]	2015	2018	2021
8	56	0.06	5.87	Shimada, et al. [26]	2016	2018	2019
9	55	0.01	4.86	Solfrizzi, et al. [27]	2017	2019	2021
10	54	0.02	6.30	Feng, et al. [28]	2017	2018	2021
11	13	0.11	4.77	Lee, et al. [29]	2011	2013	2016
12	36	0.00	16.15	Boyle, et al. [4]	2010	2013	2015
13	26	0.00	14.33	Ávila-Funes, et al. [30]	2009	2013	2014
14	48	0.01	13.43	Panza, et al. [31]	2018	2019	2021
15	35	0.03	12.27	Collard, et al. [32]	2012	2015	2017

## Data Availability

Not applicable.
